# Pulsed-field vs radiofrequency ablation for posterior wall isolation in persistent atrial fibrillation: A propensity-matched outcome analysis

**DOI:** 10.1016/j.hroo.2025.12.023

**Published:** 2026-01-10

**Authors:** Sebastian Feickert, Giuseppe D’Ancona, Kristof Biernath, Andreas A. Boehmer, Hüseyin Ince, Jasmin Ortak, Niels Christian Ewertsen

**Affiliations:** 1 Department of Cardiology and Internal Intensive Care Unit, Berlin Heart Rhythm Center, Vivantes Klinikum Am Urban, Berlin, Germany; 2 Department of Cardiology, University Medical Center Rostock, Rostock, Germany; 3 Department of Cardiology, St. Josefs-Hospital Wiesbaden, Wiesbaden, Germany; 4 Montreal Heart Institute, Montreal, Canada

**Keywords:** Persistent atrial fibrillation, Posterior wall isolation, Pulsed-field ablation, Radiofrequency, High-density mapping, Atrial substrate

## Abstract

**Background:**

Left atrial posterior wall isolation (LAPWI) is increasingly used as an adjunct to pulmonary vein isolation in patients with persistent atrial fibrillation (PerAF) despite the absence of strong evidence, but the optimal ablation modality remains uncertain.

**Objective:**

Our study focuses on procedural differences between pulsed-field ablation (PFA) and radiofrequency (RF) ablation when LAPWI is performed, rather than on the efficacy of LAPWI itself. PFA may offer advantages over RF ablation owing to its nonthermal mechanism and improved tissue selectivity.

**Methods:**

In this single-center study, 92 patients with PerAF and posterior low-voltage substrate identified using high-density mapping were treated with either PFA (n = 46) or RF ablation (n = 46) for pulmonary vein isolation plus LAPWI. Remapping was performed to confirm complete electrical isolation. Groups were 1:1 propensity score matched for analysis.

**Results:**

The primary end point was freedom from any sustained atrial arrhythmia (>30 seconds) at 12-month follow-up, assessed using structured Holter electrocardiogram monitoring at 3, 6, and 12 months. Complete LAPWI was achieved in 100% of PFA and 95.7% of RF ablation patients. Procedure time was significantly shorter with PFA (68.2 minutes [44–91] vs 86.8 minutes [58–118]; *P* < .05). Arrhythmia-free survival at 12 months was similar between groups (58.7% vs 52.2%; *P* = .57; relative risk 1.13; 95% confidence interval 0.78–1.63). Multivariable Cox regression identified LA diameter, body mass index of >30 kg/m^2^, and hypertension as independent predictors of arrhythmia recurrence. Complication rates were low and comparable.

**Conclusion:**

In patients with PerAF and posterior low-voltage substrate, PFA and RF ablation yielded similar arrhythmia-free survival at 1 year. PFA was associated with shorter procedure times and high rates of posterior wall isolation.

## Graphical abstract

**Figure:**
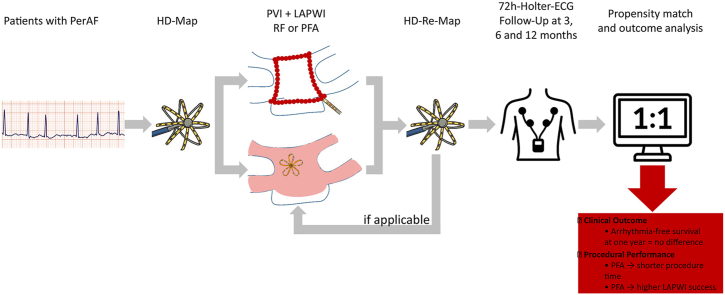

**Key Findings**

▪ Despite differences in procedural performance, arrhythmia-free survival at 12 months was similar between pulsed-field ablation (PFA) and radiofrequency (RF) ablation, indicating no difference in clinical efficacy of left atrial posterior wall isolation (LAPWI) between the 2 energy sources.
▪ PFA enabled significantly shorter procedure times than RF ablation when performing LAPWI, while maintaining a similarly high rate of acute procedural success.
▪ Both PFA and RF ablation demonstrated low complication rates and high feasibility for achieving LAPWI in patients with persistent atrial fibrillation and posterior wall low-voltage substrate.

## Introduction

Pulmonary vein isolation (PVI) represents the cornerstone of catheter ablation in patients with atrial fibrillation (AF). Although PVI alone is effective in patients with paroxysmal AF, its efficacy in persistent AF (PerAF) is limited, with long-term arrhythmia-free survival rates of approximately 60%.^1^^,^^2^ Although most empirical adjunctive ablation strategies beyond PVI have failed to show meaningful clinical benefit in PerAF,^3^, ^4^, ^5^ the recent TAILORED-AF randomized trial demonstrated that individualized, artificial intelligence–guided substrate modification may be key to improving outcomes in this population.^6^

However, the left atrial posterior wall (LAPW) has garnered particular attention because it is likely a potential driver of AF in patients with PerAF.^5^^,^^7^ Studies using high-density (HD) mapping have identified complex and low-voltage substrate in the LAPW, which may facilitate arrhythmia maintenance through mechanisms such as macroreentrant circuits and localized fibrosis. Consequently, LAPW isolation (LAPWI), in addition to PVI, has been proposed as a strategy to enhance long-term rhythm outcomes.^8^^,^^9^

The recent multicenter CAPLA randomized clinical trial^5^ showed that adding LAPWI to PVI did not improve arrhythmia outcomes in PerAF, underscoring that the clinical benefit of LAPWI remains unproven.

Traditional approaches to LAPWI using radiofrequency (RF) energy typically involve linear lesions at the roof and floor of the left atrium. However, this technique often fails to achieve durable electrical isolation of the LAPW, and lesion formation near the esophagus carries a significant risk of thermal injury, including esophageal ulceration and atrioesophageal fistula.^5^^,^^10^

Pulsed-field ablation (PFA), a novel nonthermal ablation modality based on irreversible electroporation, offers several potential advantages in this context. PFA enables tissue-selective myocardial ablation with significantly reduced risk to adjacent noncardiac structures, including the esophagus, phrenic nerves, and pulmonary veins (PVs).^1^^,^^2^^,^^11^ Early evidence suggests that LAPWI with PFA is both feasible and safe, with a high rate of durable isolation and minimal procedural complications.^1^ Moreover, PFA may offer better transmurality and lesion contiguity in LAPW ablation than RF ablation, particularly in regions with complex atrial anatomy.

Despite these potential advantages, data directly comparing the clinical efficacy of PFA and RF ablation for LAPWI in the context of PerAF are scarce, and whether the theoretical benefits of PFA translate into improved clinical outcomes remains unknown. Therefore, this study aimed to directly compare efficacy and safety outcomes in patients with PerAF undergoing PVI plus LAPWI using either PFA or RF ablation.

## Methods

### Study design and patient population

This retrospective single-center analysis included consecutive patients with PerAF who underwent first-time catheter ablation at our institution between October 2022 and June 2024. All patients underwent transesophageal echocardiography before the procedure to exclude left atrial thrombus. The inclusion criteria were the presence of PerAF and identification of low-voltage substrate (<0.5 mV) in the LAPW, as demonstrated by HD mapping using the Orion catheter (Boston Scientific) and the Rhythmia mapping system. Patients were assigned to 2 groups based on the energy source used for PVI and LAPWI: PFA or RF ablation. A 1:1 propensity score matching was performed using the nearest neighbor method based on the following variables: age, sex, body mass index (BMI), left atrial diameter, diabetes mellitus, coronary artery disease, and hypertension.

### Electrophysiological mapping

All patients underwent HD electroanatomic mapping using the Orion catheter and the Rhythmia mapping system. Voltage maps were acquired in sinus rhythm after electrical cardioversion if necessary. LAPW substrate was defined as areas with bipolar voltage of <0.5 mV. After completion of the ablation protocol, remapping was performed in all patients to assess for complete electrical isolation of both the PVs and the LAPW.

### Ablation procedure

All procedures were performed under continuous anticoagulation. After a single fluoroscopy- and pressure-guided transseptal puncture with an SL1 sheath and 71 cm needle (BRK, Abbott), intravenous unfractionated heparin was administered targeting an activated clotting time of >300 seconds.

In the PFA group, PVI and LAPWI were performed exclusively using a 31 mm pentaspline PFA catheter (Farawave, Boston Scientific) advanced through a steerable sheath (Faradrive, Boston Scientific). Each PV was treated with 4 applications in both the basket and flower configurations. LAPW ablation was achieved using overlapping applications in the flower configuration, with lateral and septal anchoring lesions delivered via placement of the guidewire into the adjacent PVs. After the initial ablation set, the Orion catheter was used to confirm electrical isolation of all PVs and the LAPW. Additional PFA applications and remapping were performed as needed to ensure complete LAPWI.

In the RF group, PVI and LAPWI were performed using a point-by-point high-power short-duration protocol with 50 W for 10 seconds using a contact force–sensing, open-irrigated RF catheter (StablePoint, Boston Scientific). The catheter was advanced through a steerable sheath (Agilis, Abbott). LAPWI was achieved by creating a roof line between the superior PVs and a posterior floor line between the inferior veins. Remapping with the Orion catheter was performed to confirm electrical isolation of all PVs and the LAPW. Additional RF lesions were delivered as necessary to close conduction gaps until complete isolation was achieved.

### Postprocedural care and follow-up

Antiarrhythmic drugs were discontinued at hospital discharge in all patients. A structured follow-up protocol was implemented, including 72-hour Holter electrocardiogram (ECG) monitoring at 3, 6, and 12 months and additional ECG or Holter monitoring in the case of arrhythmia-related symptoms.

Patients were instructed to seek medical attention and obtain ECG documentation in case of any symptoms suggestive of arrhythmia recurrence. All patients included in this analysis completed a 12-month follow-up period.

### End points

The primary end point was defined as any documented recurrence of AF, atrial flutter, or atrial tachycardia lasting ≥30 seconds after a 90-day postprocedural blanking period. Only documented episodes of atrial arrhythmias were considered for analysis.

Secondary end points included overall complications for safety and procedural measures such as procedure time, fluoroscopy time, and radiation dose.

### Statistical analysis

Continuous variables are presented as mean ± standard deviation or median with interquartile range (IQR), as appropriate. Categorical variables are expressed as counts and percentages. Comparisons between the PFA and RF groups were performed using the Student *t* test, nonparametric tests, χ^2^ test, or Fisher’s exact test, as appropriate. Cox regression analysis (backward stepwise) was performed to identify independent predictors of arrhythmia recurrence. Kaplan–Meier survival curves were constructed to estimate freedom from recurrent atrial arrhythmia at follow-up, with between-group comparisons using the log-rank test. A 2-sided *P* value of <.05 was considered statistically significant. All analyses were performed using SPSS version 28.0 (IBM Corp).

### Ethical considerations

The study was approved by the institutional ethics committee of the University of Rostock (A 2023-0213) and conducted in accordance with the Declaration of Helsinki (2024 revision).

All patients provided a written informed consent for participation in the study and for the use of their anonymized clinical and procedural data for research and publication purposes.

## Results

### Baseline characteristics

After propensity score matching (1:1 ratio), 92 patients with PerAF and low-voltage substrate at the LAPW were included in the final analysis. Of these, 46 patients underwent PVI and LAPWI using PFA (PFA group), and 46 using RF ablation (RF group). Baseline patient characteristics are presented in Table 1.

**Table 1:** Baseline characteristics

Variable	PFA group (n = 46)	RF group (n = 46)	*P* value
Age (y)	71.2 ± 8.4	69.8 ± 9.6	.459
Female sex (%)	37.0	41.3	.663
BMI (kg/m^2^)	28.3 ± 1.9	27.9 ± 2.0	.328
Diabetes mellitus (%)	15.2	15.2	1.000
Hypertension (%)	69.6	67.4	.818
Coronary artery disease (%)	17.4	17.4	1.000
Heart failure (%)	10.9	8.7	.723
Stroke/TIA (%)	4.3	2.2	.556
LA diameter (mm)	46.8 ± 4.2	46.2 ± 3.6	.464
LVEF (%)	52.0 ± 5.4	54.0 ± 4.8	.064
LVEF <40% (%)	6.5	6.5	1.000
Amiodarone (%)	6.5	10.9	.707
Flecainide (%)	4.3	2.2	.556
CHA_2_DS_2_-VASc score	4.1 ± 1.8	4.0 ± 1.6	.779

Baseline clinical characteristics of propensity score–matched patients undergoing left atrial posterior wall isolation using PFA or RF ablation. Values are presented as mean ± standard deviation or n (%). There were no statistically significant differences between groups after matching.

BMI = body mass index; LA = left atrium; LVEF = left ventricular ejection fraction; PFA = pulsed-field ablation; RF = radiofrequency; TIA = transient ischemic attack.

There were no significant differences in demographic, clinical, or echocardiographic parameters between the 2 matched cohorts. The mean age was 71.2 ± 8.4 years in the PFA group and 69.8 ± 9.6 years in the RF group. Female patients accounted for 37.0% and 41.3% of the cohorts, respectively. BMI (28.3 ± 1.9 vs 27.9 ± 2.0 kg/m^2^) and the prevalence of cardiovascular comorbidities were similar across both groups, including diabetes mellitus (15.2% vs 15.2%), arterial hypertension (69.6% vs 67.4%), coronary artery disease (17.4% vs 17.4%), heart failure (10.9% vs 8.7%), and previous stroke or transient ischemic attack (4.3% vs 2.2%), respectively.

Complete baseline characteristics of both groups are presented in Table 1.

### Procedural characteristics

All procedures were successfully completed without major intraprocedural complications. Procedural characteristics are presented in Table 2.

**Table 2:** Procedural characteristics and complications

Variable	PFA group (n = 46)	RF group (n = 46)	*P* value
Procedure duration (min), median [IQR]	68.2 [44–91]	86.8 [58–118]	<.05
Fluoroscopy time (min), median [IQR]	9.8 [5–19]	4.2 [4–11]	<.05
Fluoroscopy dose (cGy·cm^2^), median [IQR]	1990 [1250–4202]	889 [542–2120]	<.05
Complete LAPW isolation (%)	100.0	95.7	.495
Reapplications for LAPW isolation (%)	17.4	23.9	.607
Pericardial effusion (%)	0 (0.0)	1 (2.2)	1.000
Stroke/TIA (%)	0 (0.0)	0 (0.0)	n.a.
Atrioesophageal fistula (%)	0 (0.0)	0 (0.0)	n.a.
Vascular complications (%)	2 (4.3)	1 (2.2)	1.000
Coronary air embolism (%)	1 (2.2)	0 (0.0)	1.000
Phrenic nerve palsy (%)	0 (0.0)	0 (0.0)	n.a.

Procedural parameters and complication rates in patients undergoing LAPW isolation with PFA or RF ablation.

IQR = interquartile range; LAPW = left atrial posterior wall; n.a. = not available; PFA = pulsed-field ablation; RF = radiofrequency; TIA = transient ischemic attack.

Median procedure duration was significantly shorter in the PFA group than the RF group (68.2 minutes [IQR 44–91] vs 86.8 minutes [IQR 58–118]; *P* < .05; 95% confidence interval [CI] −34.9 to −2.3). Conversely, median fluoroscopy time was longer in the PFA group (9.8 minutes [IQR 5–19]) than the RF group (4.2 minutes [IQR 4–11]; *P* < .05; 95% CI 2.3–9.0). The total fluoroscopy dose was also higher in the PFA group (median 1990 cGy·cm^2^ [IQR 1250–4202] vs 889 cGy·cm^2^ [IQR 542–2120]; *P* < .05; 95% CI 384–1818).

Complete electrical isolation of the PVs and the LAPW was achieved in all patients (100%) in the PFA group and in 44 of 46 patients (95.7%) in the RF group (*P* = .495; 95% CI −1.5 to 10.2). Repeated energy applications to achieve complete LAPWI were necessary in 17.4% of patients in the PFA group and in 23.9% of patients in the RF group (*P* = .607; 95% CI −23.0 to 10.0).

Overall complication rates were low and comparable (absolute difference +2.2 percentage points; 95% CI −7.1 to 11.4) (Table 2). 1 patient (2.2%) in the RF group developed a pericardial effusion requiring percutaneous drainage. Vascular access complications occurred in 2 patients (4.3%) in the PFA group and 1 patient (2.2%) in the RF group. A single coronary air embolism (2.2%) was observed in the PFA group, without lasting clinical consequences. No atrioesophageal fistulas, phrenic nerve injuries, or strokes occurred in either group.

### Clinical outcomes

At 12-month follow-up, results showed no significant difference in freedom from any documented atrial arrhythmia (AF, atrial flutter, or atrial tachycardia >30 seconds after the blanking period) between both groups. Freedom from arrhythmia recurrence was observed in 27 of 46 patients (58.7%) in the PFA group and in 24 of 46 patients (52.2%) in the RF group (*P* = .59). Among patients with recurrence in the PFA group, 15 experienced AF and 4 had atrial tachycardia. In the RF group, 18 experienced AF and 4 had atrial tachycardia.

**Figure 1:**
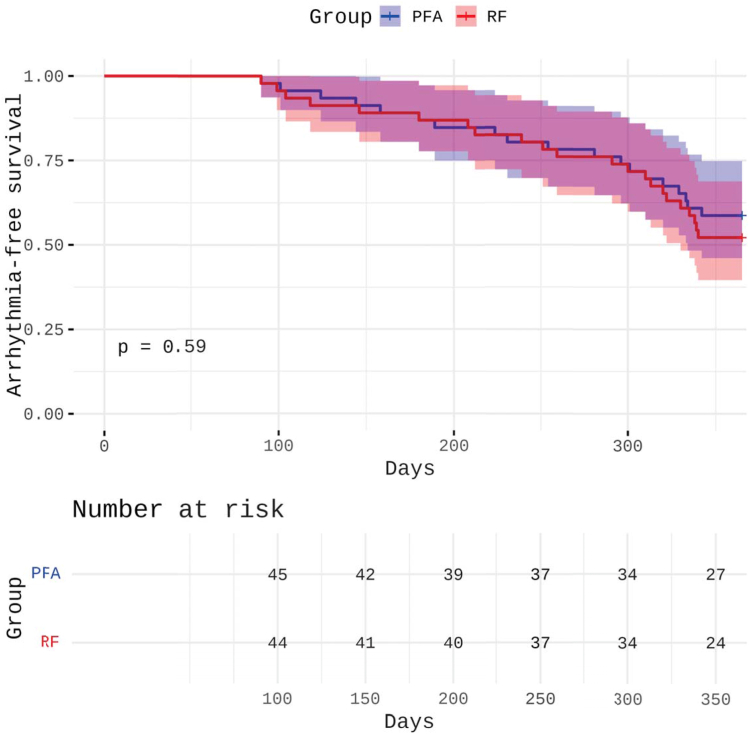
Kaplan–Meier curves illustrating freedom from any sustained atrial arrhythmia after left atrial posterior wall isolation with PFA vs RF ablation in patients with persistent atrial fibrillation and posterior low-voltage substrate. Follow-up duration was 365 days in all patients. The risk table displays the number of patients at risk at predefined time intervals. PFA = pulsed-field ablation; RF = radiofrequency.

Kaplan–Meier analysis revealed no significant difference in arrhythmia-free survival between the 2 groups over the 12-month follow-up (*P* = .59). The Kaplan–Meier at-risk table is presented in Figure 1. Most recurrences occurred within the first 6–9 months after ablation.

In multivariable Cox regression analysis, several baseline characteristics were independently associated with arrhythmia recurrence, including larger left atrial diameter (adjusted hazard ratio [HR] 1.515; 95% CI 1.033–1.797; *P* = .021), BMI of >30 kg/m^2^ (adjusted HR 1.317; 95% CI 1.033–1.679; *P* = .042), and arterial hypertension (HR 1.268; 95% CI 1.013–1.611; *P* = .038).

## Discussion

To the best of our knowledge, this is the first study to directly compare the clinical effectiveness of LAPWI using PFA vs conventional RF ablation in patients with PerAF. Although the role of posterior left atrial substrate modification remains controversial, its potential benefit in PerAF has been emphasized in recent trials, particularly in patients with low-voltage myocardium at the LAPW.^7^

Importantly, the CAPLA randomized clinical trial demonstrated no overall benefit of adding LAPWI to PVI in an unselected PerAF population.^5^ Therefore, our findings must be interpreted within this established evidence base: current data do not support routine or empirical LAPWI, even in the presence of posterior low-voltage substrate, and only a small subset of carefully selected patients is likely to derive additional benefit from this strategy.

In this propensity-matched cohort, we found no statistically significant difference in 12-month arrhythmia-free survival between PFA- and RF ablation–based LAPWI strategies, despite theoretical and procedural advantages associated with PFA. PFA allows direct electrical treatment of the entire LAPW surface with high tissue selectivity, theoretically resulting in more homogeneous and transmural lesion formation. In contrast, RF ablation–based LAPWI typically consists of linear ablation at the roof and floor of the LAPW, leaving the intervening myocardium potentially electrically.

Our findings need to be interpreted in the context of the recent CAPLA randomized clinical trial,^5^ which demonstrated no overall improvement in arrhythmia outcomes when LAPWI was added to PVI in an unselected PerAF population. In addition, the CAPLA substudy by Segan et al^12^ identified a specific electrophysiological phenotype—rapid posterior wall (PW) activity (PW cycle length <140 ms)—in which LAPWI was associated with significantly higher arrhythmia-free survival than PVI alone. These observations underscore that the clinical value of LAPWI likely depends on careful patient selection. Importantly, our study was not designed to reassess the efficacy of LAPWI but to compare procedural performance between PFA and RF ablation when LAPWI is performed in current practice. Therefore, our results should not be interpreted as evidence supporting the broader adoption of LAPWI but rather as procedural insights relevant to settings in which this strategy is selectively applied.

Despite achieving complete LAPWI in nearly all cases, the arrhythmia-free survival at 12 months in our cohort remained similar between PFA and RF ablation and was comparable with the efficacy rates historically observed for PVI alone in PerAF. These findings reinforce the growing body of evidence that empirical LAPWI does not meaningfully improve clinical outcomes beyond PVI in an unselected PerAF population.^1^^,^^5^^,^^13^^,^^14^ This point is particularly relevant given the increasing use of LAPWI in contemporary practice, often motivated by substrate-based considerations, despite limited proof of incremental benefit and a potentially higher procedural burden or risk profile than PVI alone.

This may, in part, reflect the challenge of durable lesion formation at the LAPW—particularly with thermal energy—and the limitations of current techniques in confirming sustained electrical isolation over time.

Procedurally, PFA was associated with significantly shorter procedure times, in line with previous reports highlighting its efficiency.^15^^,^^16^ This may offer practical advantages in procedural planning, patient comfort, and laboratory throughput, especially given the similar safety profile. Importantly, our study demonstrates that both PFA- and RF ablation–based LAPWI can be performed safely with low complication rates, consistent with other large-scale reports of PVI-alone strategies.

Despite the promising tissue selectivity of PFA and the completeness of LAPW coverage it enables, our results do not demonstrate a statistically superior clinical outcome compared with RF ablation–based LAPWI. This underscores the need for further randomized studies to determine whether more extensive ablation strategies beyond PVI, even if technically feasible and safe, yield clinically meaningful benefit in the context of PerAF. Notably, PFA seems to address some of the safety limitations of thermal energy—particularly the risk of esophageal injury—which remains a critical concern with LAPW ablation using RF energy.^12^

### Limitations

The retrospective, single-center design limits generalizability and may have introduced selection bias. Although propensity score matching was applied to mitigate this limitation, residual confounding cannot be excluded in the absence of randomization. Despite structured rhythm follow-ups with Holter monitoring at 3, 6, and 12 months, the lack of continuous rhythm monitoring may have resulted in missed asymptomatic or short-duration arrhythmia episodes. Furthermore, repeat electroanatomic mapping could not be performed in all patients with clinical recurrence. Consequently, we cannot determine whether LAPW reconnection contributed to arrhythmia recurrence in the RF group or whether a potentially more durable isolation achieved with PFA conferred any protective effect over time.

## Conclusion

In this propensity-matched cohort of patients with PerAF and PW low-voltage substrate, PFA- and RF ablation–based LAPWI demonstrated comparable 12-month arrhythmia-free survival. Despite shorter procedure times and complete LAPWI achieved with PFA, no significant clinical advantage was observed. Both approaches proved safe and effective, supporting the feasibility of LAPWI using different energy sources. Our findings solely describe procedural differences between PFA and RF ablation when performing LAPWI and must be interpreted in light of the CAPLA trial,^5^ which found no overall clinical benefit of LAPWI in PerAF. Still, electrophysiological phenotypes such as rapid PW activity, identified in the CAPLA substudy,^12^ may influence patient selection in future studies, which are needed to further clarify the role of adjunctive LAPWI beyond PVI in PerAF.

## Disclosures

S.F. received educational grants and a speaker’s honorarium from Boston Scientific and Biosense Webster. N.C.E. received travel grants from Boston Scientific. All other authors have no conflicts of interest to declare.

## References

[1] Reddy V.Y., Gerstenfeld E.P., Schmidt B. (2025). Pulsed field ablation for persistent atrial fibrillation: 1-year results of ADVANTAGE AF. J Am Coll Cardiol.

[2] Schmidt B., Bordignon S., Neven K. (2023). European real-world outcomes with Pulsed field ablatiOn in patients with symptomatic atrial fibrillation: lessons from the multi-centre EU-PORIA registry. Europace.

[3] Verma A., Jiang C.Y., Betts T.R. (2015). Approaches to catheter ablation for persistent atrial fibrillation. N Engl J Med.

[4] Vogler J., Willems S., Sultan A. (2015). Pulmonary vein isolation versus defragmentation: the CHASE-AF clinical trial. J Am Coll Cardiol.

[5] Kistler P.M., Chieng D., Sugumar H. (2023). Effect of catheter ablation using pulmonary vein isolation with vs without posterior left atrial wall isolation on atrial arrhythmia recurrence in patients with persistent atrial fibrillation: the CAPLA randomized clinical trial. JAMA.

[6] Deisenhofer I., Albenque J.P., Busch S. (2025). Artificial intelligence for individualized treatment of persistent atrial fibrillation: a randomized controlled trial. Nat Med.

[7] Huo Y., Gaspar T., Schönbauer R. (2022). Low-voltage myocardium-guided ablation trial of persistent atrial fibrillation. NEJM Evid.

[8] Sohns C., Bergau L., El Hamriti M. (2022). Posterior wall substrate modification using optimized and contiguous lesions in patients with atrial fibrillation. Cardiol J.

[9] Rivera A., Menezes A.S., Gewehr D.M. (2025). Adjunctive posterior wall isolation for patients with persistent atrial fibrillation: a systematic review and meta-analysis. Heart Rhythm O2.

[10] Oikawa J., Fukaya H., Wada T. (2021). Additional posterior wall isolation is associated with gastric hypomotility in catheter ablation of atrial fibrillation. Int J Cardiol.

[11] Gunawardene M.A., Middeldorp M., Pape U.F. (2025). Esophageal endoscopic findings after pulmonary vein and posterior wall isolation using pulsed field ablation - Results from the Eso-PFA study. Europace.

[12] Segan L., Chieng D., Prabhu S. (2023). Posterior wall isolation improves outcomes for persistent AF with rapid posterior wall activity: CAPLA substudy. JACC Clin Electrophysiol.

[13] Pranata R., Kamarullah W., Karwiky G., Achmad C., Iqbal M. (2024). Left atrial posterior wall isolation in addition to pulmonary vein isolation using a pentaspline catheter in pulsed-field ablation for atrial fibrillation: a systematic review and meta-analysis. Heart Rhythm O2.

[14] Gunawardene M.A., Frommeyer G., Ellermann C. (2023). Left atrial posterior wall isolation with pulsed field ablation in persistent atrial fibrillation. J Clin Med.

[15] Badertscher P., Mannhart D., Weidlich S. (2024). Left atrial posterior wall isolation using pulsed-field ablation: procedural characteristics, safety, and mid-term outcomes. J Interv Card Electrophysiol.

[16] Isenegger C., Di Bari G., Arnet R. (2025). Pulsed-Field ablation versus radiofrequency ablation in Patients undergoing Repeat Catheter ablation for atrial fibrillation. Heart Rhythm O2.

